# Transcriptomic and proteomic analyses provide insights into host adaptation of a bamboo-feeding aphid

**DOI:** 10.3389/fpls.2022.1098751

**Published:** 2023-01-11

**Authors:** Hui Zhang, Ruixun Lin, Qian Liu, Jianjun Lu, Gexia Qiao, Xiaolei Huang

**Affiliations:** ^1^ State Key Laboratory of Ecological Pest Control for Fujian and Taiwan Crops, College of Plant Protection, Fujian Agriculture and Forestry University, Fuzhou, China; ^2^ Key Laboratory of Zoological Systematics and Evolution, Institute of Zoology, Chinese Academy of Sciences, Beijing, China; ^3^ Fujian Provincial Key Laboratory of Insect Ecology, Fujian Agriculture and Forestry University, Fuzhou, China

**Keywords:** aphid-plant interaction, transcriptome, proteome, salivary protein, secretory protein

## Abstract

**Introduction:**

Salivary glands and their secreted proteins play an important role in the feeding process of sap-sucking aphids. The determination of saliva composition is an important step in understanding host plant adaptation of aphids. Pseudoregma bambucicola is a severe bamboo pest in subtropical areas and the only aphid species that can exclusively feed on hard stalks of bamboos. How this species can penetrate and degrade hard bamboo cell walls and utilize a very specialized niche are important unanswered questions.

**Methods:**

In this study, comprehensive analyses based on transcriptome sequencing, RT-qPCR, liquid chromatography-tandem spectrometry (LC–MS/MS) and bioinformatics were conducted on dissected salivary glands and secreted saliva of P. bambucicola to characterize the overall gene expression and salivary protein composition, and to identify putative effector proteins important for aphid-plant interactions.

**Results and Discussion:**

Some secretory proteins homologous to known aphid effectors important for aphid–plant interactions, such as digestive enzymes, detoxifying and antioxidant enzymes and some effectors modulating plant defenses, are also detected in salivary gland transcriptome and salivary gland and/or saliva secretomes in P. bambucicola. This indicates that these effectors are probably be essential for enabling P. bambucicola feeding on bamboo host. Although several plant cell wall degrading enzymes (PCWDEs) can be identified from transcriptome, most of the enzymes identified in salivary glands showed low expression levels and they only represent a small fraction of the complete set of enzymes for degrading cellulose and hemicellulose. In addition, our data show that P. bambucicola has no its own ability to produce pectinases. Overall, our analyses indicate that P. bambucicola may lose its own ability to express and secrete key PCWDEs, and its adaptation to unique feeding habit may depend on its symbiotic bacteria.

## Introduction

Aphids are one of the most important agricultural pest groups that feed on plant phloem sap *via* piercing-sucking mouthparts. Many aphids can also serve as vectors of plant viruses, causing serious economic damage to agriculture and forestry (Hooks and Fereres, 2006; Dedryver et al., 2010). During feeding, aphids puncture the plant epidermis using their specialized stylets that penetrate between cells and reach the phloem sieve tubes to ingest phloem sap. In their long-term coevolutionary history, plants have evolved a variety of defense systems against aphid feeding, and aphids have developed complex strategies to overcome plant defenses. As the first defensive barrier against herbivores, plant cell walls are dynamic extracellular structures composed of a thick layer of polysaccharides, such as cellulose, hemicellulose and pectin, and structural proteins (Anderson and Kieber, 2020). Aphids must first overcome the cell wall barrier of host plants to access nutrients. Plant cell wall-modifying enzymes present in aphid saliva are thought to help them penetrate the cell wall (Silva-Sanzana et al., 2020). This may be a common strategy among phloem-feeding insects for plant penetration, although the source of these enzymes may sometimes be unclear. For example, pectinase activity has been detected in secreted saliva of the *Schizaphis graminum* (Ma et al., 1990); Guo et al. (2006) has also detected pectinase and cellulase activities in saliva of *Sitobion avenae*; one putative cellulase gene sequence and several cellulase transcripts have been also identified from *Acyrthosiphon pisum* and two *Myzus* species, respectively (Watanabe and Tokuda, 2010; Thorpe et al., 2016), although there has been no protein-level validation; the *Nilaparvata lugens* can secrete a salivary endo-β-1,4-glucanase into rice plants that can degrade celluloses in plant cell walls, allowing its stylet to reach the phloem (Ji et al., 2017); and a salivary β-1,4-endoglucanase with cellulolytic activity found in sharpshooter *Homalodisca vitripennis* saliva can be secreted into plants during feeding (Backus et al., 2012).

Salivary gland is an important secretary tissue that play a crucial role during insect feeding. The insect gut also play an important role in host feeding and digestion process other than salivary glands. Enzymes in the gut of aphid, for example, are thought to be involved in the detoxification and degradation of various plant compounds (Cristofoletti et al., 2003; Matthews et al., 2010; Pyati et al., 2011; Anathakrishnan et al., 2014). For aphids, however, the salivary glands may be more important in their initial probing and feeding. Aphid salivary glands can secrete saliva containing a variety of enzymes and effectors that facilitate stylet penetration and modulate plant defense (Elzinga and Jander, 2013). Aphid saliva can be categorized into watery and gel saliva with different protein composition and function (Miles, 1959; Tjallingii, 2006). Gel saliva is secreted during the early stages of stylet penetrating and is involved in coagulation and formation of salivary sheath that can protect stylets from physical damage, while watery saliva is secreted during aphid feeding and injected into plant cells for digestion of nutrients and suppressing plant defense responses (Miles, 1999; Will et al., 2007; Will et al., 2013). The major components of gel saliva are expected to include plant cell wall-degrading enzymes (PCWDEs) facilitating stylet progress, as well as some proteins and peptides that can cause plant defense; the components of water saliva include Ca^2+^ binding proteins, proteases, detoxification enzymes and effector proteins (van Bel and Will, 2016). Several salivary effectors have been found to promote aphid feeding and plant defense suppression (Zhang et al., 2017). For example, the water-soluble salivary protein *C002*, first identified in pea aphid, has been shown to be essential for its successful feeding (Mutti et al., 2008), and overexpression of *MpC002* in *Myzus persicae* on *Nicotiana benthamiana* could promote aphid fecundity (Bos et al., 2010). Some other effectors such as *Me10* and *Me23* in *Macrosiphum euphorbiae*, as well as *Mp1*and *Mp2* in *M. persicae* have also been proved to enhance aphid fecundity or promote aphid colonization (Atamian et al., 2013; Pitino and Hogenhout, 2013).

The composition of salivary proteins is supposed to be a key factor limiting aphids’ host range (Elzinga and Jander, 2013). Characterization of salivary components is crucial for understanding adaptation of aphids to specific host plants. Salivary proteins are generally identified by transcriptomic and/or proteomic analyses of the dissected salivary glands and/or secreted saliva (Nicholson et al., 2012; Atamian et al., 2013; van Bel and Will, 2016; Zhang et al., 2017; Dommel et al., 2020; Zhang et al., 2020). Integrated transcriptomic and proteomic analyses of salivary protein composition can help identify new salivary protein, and obtain a more accurate and comprehensive salivary protein repertoire. The salivary protein composition has been investigated by integrated omics analysis in some aphid species, such as the *A. pisum* (Carolan et al., 2011).

Among the over 5,100 aphid species, the social aphid *Pseudoregma bambucicola* is the only one exclusively feeding on hard stems of bamboos. This species is mainly distributed in subtropical Asian areas and exclusively specialized on *Bambusa* bamboos. Bamboo is known to have an enhanced mechanical hardness and highly lignified and fibrotic cell walls. The secondary wall structure of bamboo fiber shows unique multilayered structure (Preston and Singh, 1950). Moreover, the cell wall porosity of bamboo is generally lower than that of wood species (Cao et al., 2022). But how *P. bambucicola* stylet can penetrate bamboo cell walls remains largely unknown, and answering this question is crucial for understanding the mechanisms underlying high specialization of feeding niche. We need to explore this question in two aspects: on the one hand, we need to know the role of *P. bambucicola* itself in its unique feeding niche and diet specialization; on the other hand, the contribution by the symbiotic partners to host adaption in *P. bambucicola* should also be explored in parallel. We are actively working on resolving these issues. Previous studies on the symbiotic bacterial community of *P. bambucicola* have indicated that this aphid harbours symbiotic *Pectobacterium*, which may produce PCWDEs and assist *P. bambucicola* in feeding hard bamboo stems (Charkowski et al., 2012; Liu et al., 2021; Liu et al., 2022). However, from this aphid’s perspective, it is unclear what is its salivary protein composition and what role itself can play in breaking through the plant cell wall barrier during its feeding.

In this study, a comprehensive analysis based on transcriptome and liquid chromatography-tandem spectrometry (LC–MS/MS) was conducted on dissected salivary glands and secreted saliva of *P. bambucicola* to characterize the overall gene expression and salivary protein composition, and to identify putative effector proteins important for aphid-plant interactions. This study can promote our understanding for the role of salivary glands in host specialization of *P. bambucicola*, and provide insights into its adaptation to unique feeding habit.

## Materials and methods

### Aphid collection and sample preparation

Parthenogenetic adults of *P. bambucicola* used in this study were collected from Fuzhou, China in 2020. Paired salivary glands and guts of *P. bambucicola* were dissected in ice-cold phosphate-buffered saline solution (PBS, 10 mM NaH_2_PO4, 1.8 mM KH_2_PO4, 140 mM NaCl and 2.7 mM KCl, pH=7.4) using fine tweezers. The dissected tissues were quickly washed twice in PBS solution and immediately snap-frozen in liquid nitrogen, and then stored in a -80°C freezer. Approximately 200 pairs of salivary glands were used for transcriptome and proteome sequencing, respectively, and 200 pairs of salivary glands and 150 guts were also prepared for RT-qPCR for comparison of gene expression between samples. Each sample consisted of three biological replicates. Most previous studies used artificial diets to collect saliva and identified aphid salivary proteins successfully (Harmel et al., 2008; Carolan et al., 2009; Rao et al., 2013; Yang et al., 2018). However, it is difficult to rear *P. bambucicola* with traditional artificial diet as other aphids due to its unique feeding habitat. Therefore, we turned to identify injected salivary proteins by comparative proteomic analysis of fed and unfed bamboos with aphid salivary gland transcriptome data as the search database. For identification of injected salivary proteins by *P. bambucicola*, the *Bambusa multiplex* stems that had been fed continuously by over thousands of *P. bambucicola* individuals for three days were collected, with stems that had not been fed using for control. Several samples were unqualified and thus discard during the sequencing process, two bamboo samples fed and one sample unfed by *P. bambucicola* were used finally. Transcriptome and proteome sequencing were performed by Sangon Biotech (Shanghai, China) and Jingjie PTM BioLab (Hangzhou, Zhejiang, China), respectively.

For identifying all PCWDEs in *P. bambucicola* whole body, individuals of different morphs and developmental stages, including newborn 1st instar normal nymphs, newborn 1st instar soldiers, older 1st instar normal nymphs, older 1st instar soldiers, medium instar normal nymphs, viviparous adult females producing soldiers and viviparous adult females producing normal nymphs, were also collected and subjected for transcriptome sequencing.

### Transcriptome sequencing and RT-qPCR analysis

The *P. bambucicola* samples across different morphs and different developmental stages and approximately 200 pairs of dissected salivary glands of *P. bambucicola* were used for RNA extraction using TRIzol Reagent (Qiagen, CA). The RNA concentration and quality were assessed by a NanoDrop spectrophotometer, gel electrophoresis and an Agilent 2100 Bioanalyzer system (Agilent Technologies, CA, USA). Qualified RNA was then used for cDNA library construction. The generated libraries were sequenced using the DNBSEQ sequencing platform. The obtained raw data was subjected to removing adapters, low quality sequences and ambiguous nucleotides (reads with more than 5% N bases). The obtained clean data was used for *de novo* assembly with the Trinity (Grabherr et al., 2011) to obtain final unigenes. Bowtie (Bowtie, RRID: SCR_005476) (Langmead and Salzberg, 2012) was used for aligning clean reads to the unigene library, and then RSEM (RSEM, RRID: SCR_013027) (Li and Dewey, 2011) was used to calculate the gene expression level of unigenes. The relative abundance of unigenes was measured by FPKM, which represents fragments per kilobase of transcript per million mapped reads. For functional annotation, all predicted unigenes were run blast against multiple public databases, including non-redundant protein (Nr) database, Nt, Swiss-Prot, Kyoto Encyclopedia of Genes and Genomes (KEGG), euKaryotic Ortholog Groups (KOG), Pfam and Gene Ontology (GO) databases.

Insects can adapt to plant defense responses by utilizing effectors from a variety of sources, such as salivary proteins, intestinal proteins and symbiotic microorganism derived functional proteins (Zhao et al., 2019). A total of 11 genes associated with aphid feeding, including five genes related to digestion and six genes related to defense, were randomly selected for detection of gene expression levels between salivary glands and guts, with the HSP70A1 (heat shock protein 70 A1-like) and MGST1 (microsomal glutathione S-transferase 1-like) used as reference genes to normalize selected genes’ expression (**Table S1**). Primer Premier 5.0 (Premier Biosoft, CA, USA) was used to design RT-qPCR specific primers for selected and reference genes, as shown in **Table S1**. cDNA was synthesized using FastKing gDNA Dispelling RT SuperMix (Tiangen, Beijing, China). RT-qPCR was performed with Green qPCR SuperMix Kit (TransGen Biotech, Beijing, China) following the manufacturer’s instructions. Three biological replicates were performed on each salivary gland and intestinal tract sample, and each biological replicate was run in three technical replicates. All data were analyzed by Graphpad (GraphPad, RRID: SCR_000306) (http://graphpad.com/) version 9.0 software with unpaired t-test (*P* < 0.05).

### LC-MS/MS analysis of salivary glands and saliva

The label-free LC-MS/MS quantitative proteomic analysis was performed by the Jingjie PTM BioLab. The salivary gland samples were grinded with liquid nitrogen into cell powder and transferred to 5 ml centrifuge tube. After adding four times the volume of lysis buffer (including 1% SDS and 1% protease inhibitor cocktail), the cell powder samples were boiled with a metal bath at 95°C for 10min, and were sonicated with a high intensity ultrasonic processor (Scientz, Ningbo, China). To remove cell debris, the protein solution was spun for 10 min (12000 g at 4°C) and the supernatant was pipetted into clean tubes. The protein concentration was determined using BCA Protein Assay kit (Beyotime, Shanghai, China) following the manufacturer’s instructions.

For digestion, an equal amount of protein for each sample was used and lysis buffer was added to adjust to the same volume. After adding dithiothreitol (DTT) to a final concentration of 5 mM, the protein solution was incubated at 56°C for 30 min, followed by adding iodoacetamide (IAA) to 11 mM final concentration and incubating 15 min at room temperature in the dark to alkylate cysteines. The alkylated protein samples were transferred to ultrafiltration tubes, centrifuged at 12000 g for 20 min at room temperature. The protein was re-suspended in 8 M urea (Sigma) for 3 times, and then urea was also re-suspended with 100mM ammonium bicarbonate solution for 3 times. Trypsin was added for a final trypsin:protein ratio of 1:50 (w/w) and incubated overnight. The peptides were recovered by centrifugation at 12000 g for 10 min at room temperature, and then recovered again with ultrapure water. The two peptide solutions were then combined.

The peptides were dissolved with solvent A (0.1% formic acid and 2% acetonitrile in water) and then separated using the Easy-nLC 1200 ultra-high-performance liquid system. The separated peptides were ionized by injection into an NSI ion source and then analyzed by Orbitrap Exploris™ 480 mass spectrometers (Exploris 480, Thermo Fisher Scientific, USA). The electrospray ionization voltage was set to 2.3 kV, and a high-resolution Orbitrap was used to detect and analyze the peptide parent ions and their secondary fragments. The primary mass spectrum range was 400-1200 m/z with the scanning resolution was set to 60000. The fixed start point of the secondary mass spectrum scan range was 110 m/z with the scanning resolution of 15000, and TurboTMT was set to Off. A data dependent scanning (DDA) program based on Cycle time was used as the data acquisition mode. Specifically, within a 1.0-s cycle period, the parent ions of the peptide were selected according to the sequence of the signal intensity from high to low, and then entered the HCD collision pool to fragment with 27% of the fragmentation energy. The secondary mass spectrometry analysis was also performed sequentially. To improve the efficient utilization of MS, the automatic gain control (AGC) was set to 100%, the signal threshold was set to 5E4 ions/s, the maximum injection time was set as Auto, and the dynamic exclusion time of tandem MS scanning was set to 20 s to avoid repeated parent ion scanning.

The resulted MS/MS data were analyzed using Maxquant search engine (version v1.6.15.0) (Prianichnikov et al., 2020) with the protein sets (22,597 sequences) of salivary gland transcriptome using as the retrieval database and an inverse decoy library used to calculate the false positive rate (FDR). The cleavage enzyme was Trypsin/P allowing up to 2 cleavages; the minimum peptide length was 7 amino acid residues; the maximum number of modifications of the peptide was set as 5; the mass tolerance of precursor ions was 20 ppm in the first search and 4.5 ppm in the main search, respectively. The mass tolerance of fragment ions is 20 ppm. Carbamidomethyl on cys was set as a fixed modification with oxidation on Met, acetylation on protein N-terminal and decarboxamidation as variable modifications. The false discovery rate (FDR) for both protein identification and peptide−spectrum matches (PSMs) identification was 1%.

For protein extraction of bamboo tissues, samples of bamboo fed and unfed by *P. bambucicola* were grinded with liquid nitrogen. The powder samples were sonicated with a high intensity ultrasonic processor (Scientz, Ningbo, China) after adding four times the volume of lysis buffer (including 10 mM dithiothreitol and 1% protease inhibitor cocktail). An equal volume of Tris-saturated phenol was then added and centrifuged for 10 min (5500 g at 4°C). The supernatant was collected in clean centrifuge tubes and five times the volume of 0.1 M ammonium acetate/methanol were added and incubated at -20°C overnight. After centrifugation at 4°C for 10 min, the supernatant was removed, and the precipitate was washed with cold methanol once and cold acetone for three times, respectively. The precipitate was redissolved with 8 M urea (Sigma), and the protein concentration was determined using BCA Protein Assay kit (Beyotime, Shanghai, China) following the manufacturer’s instructions. For enzymatic digestion, an equal amount of protein for each sample was taken and adjusted to the same volume with lysis buffer. TCA was added slowly to a final concentration of 20% TCA, mixed by vortex, and precipitated for 2h at 4°C. After centrifugation at 4500g for 5min, the supernatant was discarded and the precipitate was washed with precooled acetone for two to three times. After the precipitation was dried, TEAB was added to a final concentration of 200 mM, and the precipitation was broken up by ultrasound. Trypsin was then added at 1:50 (trypsin: protein, m/m) ratio for digestion overnight. Dithiothreitol (DTT) was added to make the final concentration of 5 mM and reduced at 56°C for 30 min. Then iodoacetamide (IAA) was added to 11 mM final concentration and incubated for 15 min at room temperature under in the dark. The subsequent LC-MS/MS analysis were then performed as described above. And the obtained MS/MS spectra data were searched separately against the salivary gland transcriptomic database and *Bambusa* protein database (including 14361 proteins) download from Nr protein database of the NCBI (accessed on April 19, 2022).

The identified salivary gland proteins, saliva proteins and bamboo proteins was annotated with multiple public databases, including Nr, KEGG, Swiss-Prot, Pfam, GO and KOG databases. Functional enrichment analysis was then conducted with the functions phyper in R software, with FDR adjust *P*-value (Qvalue) < 0.05 as the threshold.

### Bioinformatic analysis

Aphid effectors are likely secreted proteins delivered into the saliva secreted by salivary glands to mediate plant defenses (Bos et al., 2010). For identification of candidate effectors, signal peptides were predicted from the amino acid sequences of dual transcriptomic-proteomic data from salivary glands as well as proteomic data from saliva using SignalP (SignalP, RRID: SCR_015644) (https://dtu.biolib.com/SignalP-6) v6.0 (Teufel et al., 2022), followed by DeepTMHMM (Hallgren et al., 2022) to identify transmembrane domains for proteins containing signal peptides. Proteins containing an N signal peptide but no transmembrane domain were regarded as candidate effectors.

The degradation of plant cell wall components requires a large repertoire of highly specialized carbohydrate-active enzymes (CAZymes) that are produced by the organism itself or its associated symbiotic microbes (Ni and Tokuda, 2013; Scully et al., 2013; Bredon et al., 2019). Firstly, Hmmscan program in the HMMER (Hmmer, RRID : SCR_005305) (http://hmmer.janelia.org/) version 3.1b2 (Eddy, 1998) was used to search amino acid sequences of transcriptome and proteome against the family specific HMM profiles of CAZymes within dbCAN HMMdb v11 to identify CAZymes and assign them to CAZy families, with an e-value cutoff 1e-3 (≤80 aa) or 1e-5 (>80 aa) and coverage above 30% as the filter threshold. CAZy families can be classified into glycoside hydrolases (GHs), glycosyltransferases (GTs), polysaccharide lyases (PLs), carbohydrate esterases (CEs), auxiliary activities (AAs) and carbohydrate-binding modules (CBMs) (Drula et al., 2022). Enzymatic activity of all identified CAZymes were detected using Hotpep (Busk et al., 2017) to determine whether they are candidate plant cell wall degrading enzymes (PCWDEs). The identified candidates were further confirmed as putative PCWDEs by reference to Tokuda (2019). The transcriptome data used for PCWDEs identification in this study include salivary gland transcriptome and whole-body transcriptome of *P. bambucicola* across different morphs and developmental stages.

## Results

### Transcriptome overview of SG of *Pseudoregma bambucicola*

**Figure 1:**
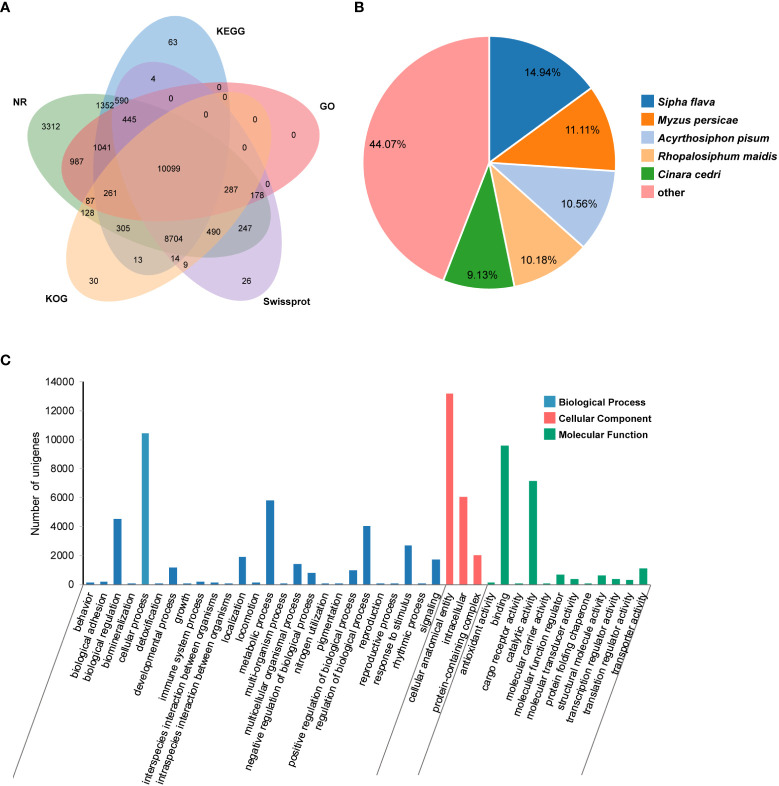
Transcriptomic annotation for *Pseudoregma bambucicola* salivary glands. **(A)** Venn diagram of annotated results *via* different databases. **(B)** Species distribution of annotation results in Nr database. Only the top five closely matched species are shown. **(C)** Gene Ontology classification of *Pseudoregma bambucicola* salivary gland unigenes.

An average of 47,326,665 bp raw reads was yielded from transcriptome of *P. bambucicola* salivary glands. After data filtering, a total of 19.47 Gb clean data was used for *de novo* assembly, resulting in 48,028 unigenes with an average length of 1,310 bp and N50 of 2,400 bp. There were 28,512 (59.37%), 24,816 (51.67%), 21,093 (43.92%), 22,891 (47.66%), 20,427 (42.53%), 20,570 (42.83%) and 13,385 (27.87%) unigenes homologous to known sequences in the Nr, Nt, SwissProt, KEGG, KOG, PFAM and GO databases, respectively. About 32,111 (66.86%) unigenes were functionally annotated in at least one of the used databases, and many of them could be annotated by multiple databases (**Figure 1A**). The unigenes showed the most similarity with *Sipha flava* according to the matched species distribution of annotation based on Nr database (**Figure 1B**).

GO function classification was conducted on predicted unigenes and showed that most of them were enriched in cellular anatomical entity, cellular process, binding, catalytic activity and metabolic process (**Figure 1C**). The top 20 highly-expressed unigenes include some genes associated with mitochondrial activity, genes encoding a invertebrate-type lysozyme 6, a odorant-binding protein 2, a prohormone-2, a alpha-N-acetylgalactosaminidase, a putative sheath protein and some genes encoding proteins with unknown functions (**Table S2**). These highly expressed genes in salivary glands such as the gene encoding putative sheath protein and genes with unknown functions were worth for further study. In addition, many homologous genes encoding salivary proteins that are known to paly an important role in aphid-plant interactions, such as some digestive enzymes, detoxifying enzymes, antioxidant enzymes and some effector proteins modulating plant defenses, were also identified in *P. bambucicola* salivary glands (**Table S3**). These results suggest that some salivary components are conserved across different aphids.

### RT-qPCR analysis of feeding–related genes

**Figure 2:**
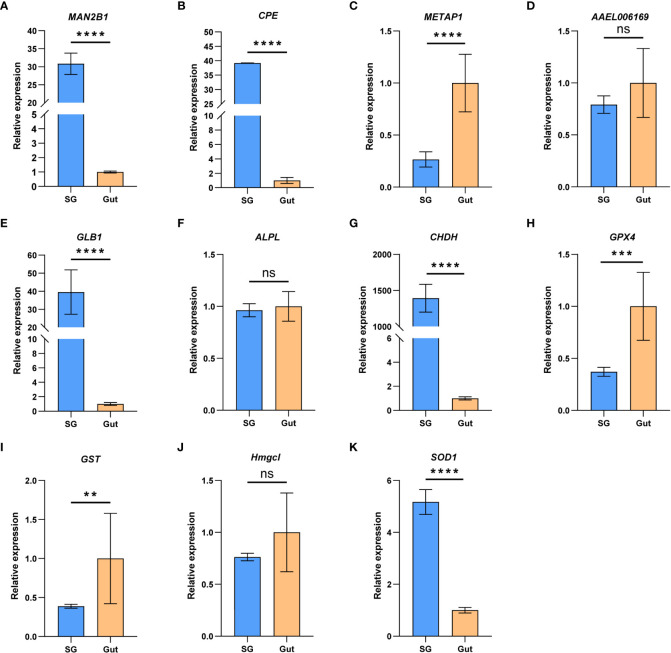
The relative gene expression of the 11 feeding-related genes between salivary glands (SG) and gut (Gut) of *Pseudoregma bambucicola* detected by RT-qPCR. **(A)** lysosomal α-mannosidase (*MAN2B1*); **(B)** carboxypeptidase E like (*CPE*); **(C)** methionine aminopeptidase-related gene (*METAP1D*); **(D)** lysosomal aspartic protease (AAEL006169); **(E)** β-galactosidase (*GLB1*); **(F)** alkaline phosphatase (*ALPL*); **(G)** glucose dehydrogenase (*CHDH*); **(H)** phospholipid hydroperoxide glutathione peroxidase (*GPX4*); **(I)** glutathione S-transferase (*GST*); **(J)** hydroxymethylglutaryl-CoA lyase (*Hmgcl*); **(K)** superoxide dismutase (*SOD1*). Heat shock protein 70 A1 and microsomal glutathione S-transferase 1 were used as internal reference genes. Asterisks above the bars indicate significant differences (**P < 0.01; ***P < 0.001; ****P < 0.0001). “ns” indicates not significant (P > 0.05).

In addition to the salivary glands, guts are also important for insect feeding. To understand the relative role of salivary gland and gut in *P. bambucicola* feeding, expression levels of 11 genes, including five genes related to digestion and six genes involved in detoxification and antioxidant activities, were detected between salivary glands and guts of *P. bambucicola* by RT-qPCR. All 11 genes expressed in both salivary glands and guts. Among the five digestive-related genes, significant differences were found in the expression of beta-galactosidase-like (*GLB1*), lysosomal alpha-mannosidase (*MAN2B1*), carboxypeptidase E-like (*CPE*) and methionine aminopeptidase 1D, mitochondrial (*METAP1D*) between salivary glands and guts except the AAEL006169 (lysosomal aspartic protease), among which the *GLB1*, *MAN2B1* and *CPE* showed evidently higher expression levels in salivary glands (**Figures 2A–E**). For six genes involved in detoxification and antioxidant activities, the expression level of glucose dehydrogenase [FAD, quinone] (*CHDH*) in salivary glands was remarkably higher than that in guts; and superoxide dismutase [Cu-Zn] 1 (*SOD1*) also showed higher expression levels in salivary glands. Phospholipid hydroperoxide glutathione peroxidase (*GPX4*) and glutathione S-transferase-like (*GST*) were highly expressed in guts compared with salivary glands (**Figures 2F–K**). These results suggest that both salivary glands and guts may play important roles in digestion and detoxification in *P. bambucicola* plant feeding.

### Proteins identified from salivary gland and saliva

**Figure 3:**
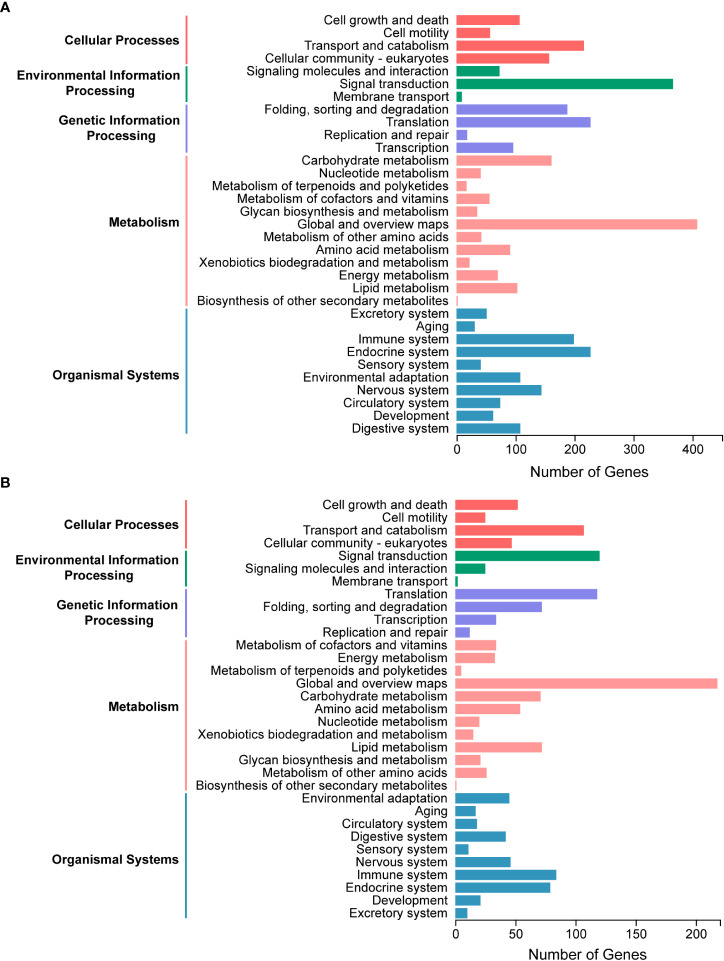
Kyoto Encyclopedia of Genes and Genomes (KEGG) pathway analysis of salivary components in *Pseudoregma bambucicola*. **(A)** Functional classification of metabolic pathways for salivary gland proteins. **(B)** Functional classification of metabolic pathways for putative saliva proteins secreted into bamboos.

A total of 4793 proteins were detected from the salivary gland proteome. Of them, 3115 proteins attributed to at least one GO term, with the cellular metabolic process and organic substance metabolic process, organelle and cytoplasm, protein binding and hydrolase activity being the two most represented terms in each of the three categories, respectively (**Figure S1A**). Proteins without unique peptides in two of three replicates and those with an average of unique peptides less than two were filtered out, resulting in 2442 candidate proteins. All proteins were annotated with the KEGG pathway database to characterize the general metabolic functions of the salivary gland proteome, and many proteins were classified and associated with global and overview maps, signal transduction, endocrine system, translation, and transport and catabolism pathways (**Figure 3A**), consistent with the biological roles of salivary glands.

The raw MS/MS data of bamboo tissues unfed and fed by aphids were then analyzed and searched against the transcriptomic data of *P. bambucicola* salivary glands to identify putative salivary proteins in secreted saliva. In total, 3244 proteins were detected, and 1496 of them were expressed only in bamboo tissues after aphid feeding, and were regarded as aphid salivary candidates secreted into host plants. To reduce false positives, 1160 proteins with at least two unique peptides were selected for further metabolic functional analysis. The majority of proteins participated in pathways of global and overview maps, signal transduction, translation, transport and catabolism and immune system, which was generally similar to that of salivary gland proteome (**Figure 3B**).

KEGG pathway enrichment analysis showed that the salivary gland proteins were significantly enriched in pathways of proteasome, protein processing in endoplasmic reticulum, carbon metabolism, citrate cycle (TCA cycle), oxidative phosphorylation and protein export, while ribosome and oxidative phosphorylation were the most representative pathways for putative saliva proteins (**Figure S1B, C**). These results may indicate the important roles of salivary glands in protein secretion and energy metabolism.

### Salivary secretory proteins

From transcriptome data of salivary glands, 1213 putative secretory proteins were predicted. Annotation against NCBI Nr database showed that 812 (66.94%) of all identified secretory proteins were functionally annotated, 359 (29.60%) proteins were annotated with unknown functions, and 42 (3.46%) proteins showed no similarity with all known sequences in the Nr database (**Table S4**). Functional enrichment analysis of these putative secretory proteins showed that the most enriched GO terms were structural constituent of cuticle, extracellular region and carbohydrate metabolic process, and the most enriched KEGG pathways were RNA polymerase, lysosome and other glycan degradation (**Figure S2**).

When we identified secreted proteins from mass spectrometry proteins, a total of 196 and 114 putative secreted effector candidates were predicted from the salivary gland and saliva proteome of *P. bambucicola*, respectively. Many of them were hypothetical proteins with unknown functions or functionally annotated proteins whose roles in aphid-plant interactions are not clear. Some candidate secretory effectors were homologous proteins also characterized in secretome of other aphid species, which had been supposed to play important roles in aphid-host interactions. For example, some detoxifying and antioxidant enzymes including glucose dehydrogenase, glutathione S-transferase, several carboxylesterases and peroxidases, were identified from salivary gland or saliva of *P. bambucicola* (**Table S5**). The putative salivary secretory effectors also contained some digestive enzymes such as sugar-degrading enzymes, carboxypeptidase, cathepsins, serine proteases and phospholipase, and some effectors modulating plant immunity and defense such as apolipophorin, odorant binding protein and yellow-like protein. In addition, some salivary glue proteins and cuticle proteins were also identified in both salivary gland and saliva of *P. bambucicola*, while two sheath proteins were detected only in salivary glands (**Table S5**). There were 44 putative secretory effectors in both salivary gland and saliva, including three salivary glue protein, a sheath protein (mucin-5AC protein), a venom serine carboxypeptidase, cathepsin L, phospholipase A-2-activating protein, odorant binding protein in addition to the above mentioned common salivary gland protein and several cuticle proteins. These putative secretory effectors may help promote aphid stylet penetration, digestion and detoxification activities, or contribute to suppression or activation of plant defense responses.

### Plant cell wall degrading enzymes

**Figure 4:**
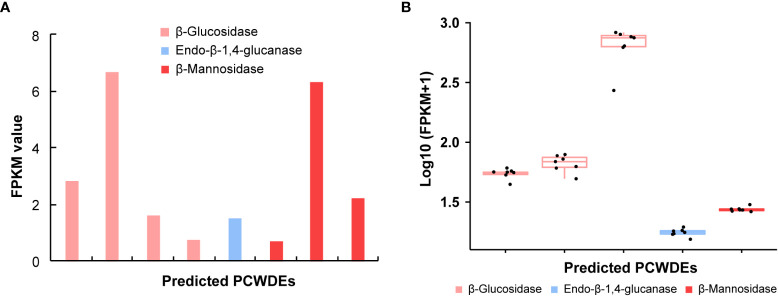
The expression patterns of genes encoding plant cell wall-degrading enzymes (PCWDEs) in different samples of *Pseudoregma bambucicola*. **(A)** The expression pattern of different PCWDEs identified from salivary glands. The bars from left to right in the histogram represent β-glucosidase 1, β-glucosidase 4, β-glucosidase 5, β-glucosidase 6, endo-β-1,4-glucanase 1, beta-mannosidase 2, beta-mannosidase 3 and beta-mannosidase 1, respectively (see **Table S6** for details). **(B)** The expression pattern of PCWDEs across different morphs or developmental stages. The boxplots from left to right represent β-glucosidase 1, β-glucosidase 2, β-glucosidase 3, endo-β-1,4-glucanase 1 and beta-mannosidase 1, respectively. The black dots in each box in figure B represent *Pseudoregma bambucicola* of different morphs or developmental stages, including newborn 1st instar soldiers, older 1st instar soldiers, newborn 1st instar normal nymphs, older 1st instar normal nymphs, middle-stage normal nymphs, soldier producing adults and normal nymph producing adults.

The PCWDEs from the transcriptome of whole body and salivary glands were identified (**Table 1**, **Table S6**). A total of eight potential PCWDEs were identified based on salivary gland transcriptome, including four β-glucosidases, one endo-β-1,4-glucanase and three β-mannosidases. However, most of genes encoding PCWDEs showed very low expression levels (**Figure 4A**, **Table 1**). We did not identify any transcripts with potential pectinase activity, indicating that the *P. bambucicola* may loss ability to secrete and degrade pectin. When we detected PCWDEs expressed in translational levels using the proteome data of salivary glands and secreted saliva, only one β-glucosidase and one β-mannosidase were detected at protein level in salivary gland, and no other PCWDEs were detected at protein level in the secreted saliva except for four β-glucosidases with putative cellulolytic activity. Of these, only one β-mannosidase identified from salivary gland proteome and one β-glucosidase from saliva proteome were predicted containing secretory signal. Besides, two of the four β-glucosidase transcripts identified in the proteome of secreted saliva were not full-length and so it is uncertain whether they contain secretory signal or not.

**Table 1:** Plant cell wall-degrading enzymes (PCWDEs) candidates identified from the whole body and salivary gland transcriptomes of *Pseudoregama bambucicola*.

Enzyme name	EC number	CAZy family	Number of PCWDEs		Potential secreted PCWDEs	
			Whole body	Salivary glands	Whole body	Salivary glands
Cellulases						
β-Glucosidase	3.2.1.21	GH1	3	4	1	1
Endo-β-1,4-glucanase	3.2.1.4	GH9	1	1	1	1
Hemicellulases						
β-Mannosidase	3.2.1.25	GH2	1	3	1	3

EC number, Enzyme Commission number; CAZy family, Carbohydrate-Active Enzymes family, See **Table S6** for overlapping or unique identification of CAZymes between samples.

To obtain more complete PCWDEs in *P. bambucicola*, PCWDEs from whole-body transcriptome across different morphs and developmental stages of *P. bambucicola* were also identified. A total of three β-glucosidases, one endo-β-1,4-glucanase and one β-mannosidase were identified, among which β-glucosidase 1, the endo-β-1,4-glucanase 1 and beta-mannosidase 1 were found in both the whole-body and salivary gland (**Table 1**, **Table S6**). Except for two glucosidases (β-glucosidase 2 and β-glucosidase 3) found only in whole-body, the three putative PCWDEs identified in both the whole-body and salivary gland all contained secretory signals. All predicted PCWDEs from body transcriptome of *P. bambucicola* exhibited high expression levels (with an average of FPKM > 16) (**Figure 4B**).

### Changes of bamboo proteins in response to aphid feeding

An comparative proteomic analysis of bamboo tissues unfed and fed by aphids may reveal the changes in protein expression and plant cellular process modulated by *P. bambucicola*. A total of 171 proteins were differentially expressed between two types of bamboo tissues, with 71 of them up-regulating and 100 of them down-regulating in bamboo after being fed respectively (**Table S7**). The highly expressed proteins were mainly enriched in categories of non-membrane-bounded organelle, nucleolus, cell surface, hydrolase activity and maintenance of protein location in cell, while the downregulated proteins were mainly associated with vesicle, golgi apparatus, cellulose synthase activity and plant cell wall biogenesis (**Figure S3**).

## Discussion

Aphid salivary glands can secrete saliva containing a variety of effectors that is important for aphid-plant interactions. In this study, combined transcriptomic and mass spectrometry (LC-MS/MS) analyses were conducted on salivary glands and secreted saliva of *P. bambucicola* to get a more comprehensive understanding of the salivary composition and the role of salivary glands in its successful feeding on the hard bamboo stalks. Transcriptome analysis of salivary gland components showed that many genes are abundant in binding, catalytic activity and metabolic process, and several mitochondrial genes associated with energy metabolism are especially highly expressed, suggesting that salivary glands have strong and active catalytic and energy metabolic activities. This is consistent with the biological characteristics and functions of salivary glands and salivary components. Many homologous salivary proteins important for aphid–plant interactions, such as digestive enzymes, detoxifying and antioxidant enzymes and some effectors modulating plant defenses (van Bel and Will, 2016; Zhang et al., 2017) are also detected in *P. bambucicola* salivary glands based on deduced amino acid sequences, suggesting that some similar strategies may employed by phloem-feeding aphids to overcome plant defenses.

We also detected and compared the expression of 11 genes encoding salivary proteins between salivary glands and guts, which are thought to be involved in aphid-plant interactions. The expression of transcripts for digestive enzymes including beta-galactosidase, lysosomal alpha-mannosidase, carboxypeptidase E, detoxifying enzyme glucose dehydrogenase and the antioxidant enzyme superoxide dismutase show much higher expression levels in salivary glands than in guts. Beta-galactosidase, a member of glycosyl hydrolase family that is involved in the hydrolysis of carbohydrates, has also been detected in *S. avenae*’s saliva (Rao et al., 2013). While the general role of lysosomal alpha-mannosidase in insects has been poorly characterized, a homolog of it is also found to be highly expressed in salivary glands of *Diaphorina citri* (Wu et al., 2021), suggesting an important role in interactions between phloem-feeing insects and host plants. Carboxypeptidases are important digestive enzymes and the carboxypeptidase E is an insect neuropeptide processing enzyme regulating secretory pathway, and is required for the biosynthesis of pheromone and neuropeptide (Stone et al., 1994). The carboxypeptidase E has been assumed to be present only in brain cells producing peptidic hormones, while its high expression found in *P. bambucicola* salivary glands may imply an important role in feeding and digesting plants. During aphid feeding, plants can produce a variety of toxic chemicals and defensive compounds against aphids. While aphids also have some detoxifying enzymes for suppression of plant defenses. The glucose dehydrogenase belongs to the GMC oxidoreductase family and members of this family were shown to be present in caterpillar saliva most likely suppressing plant defenses by transcript regulation (Bede et al., 2006). Glucose dehydrogenase has been previously characterized in several other aphids, such as the *A. pisum* (Carolan et al., 2011), *Diuraphis noxia* (Nicholson et al., 2012), *S. graminum* (Zhang et al., 2022), *S. avenae* (Zhang et al., 2017), *M. euphorbiae* (Chaudhary et al., 2015), *Metopolophium dirhodum* (Rao et al., 2013) and *Schlechtendalia chinensis* (Yang et al., 2018). The dramatically overexpression of this gene in the *P. bambucicola* salivary glands may indicate that it also plays an important role in *P. bambucicola* feeding and adaption to bamboo. Superoxide dismutase can destroy toxic radicals and protect insect from the plant ROS damage and has been also reported in other aphids (Lukasik, 2007). The highly expressed superoxide dismutase in *P. bambucicola* salivary gland may involved in scavenging ROS induced by plant defense responses. Collectively, these notably highly expressed genes in salivary glands may play an important role in detoxifying phytochemicals and successful feeding on bamboo hosts. However, genes encoding the digestive enzyme methionine aminopeptidase 1D, antioxidant enzyme phospholipid hydroperoxide glutathione peroxidase, and detoxifying glutathione S-transferase expressed at higher levels in the gut than in salivary glands, suggesting the importance of guts in digestion and detoxification during plant feeding in *P. bambucicola*.

Salivary components of *P. bambucicola* were also characterized at the protein level from the dissected salivary glands and secreted saliva by LC–MS/MS analysis. Due to its special feeding habitat, it is difficult to simulate the feeding process of *P. bambucicola* and collect saliva *via* artificial diet as in other aphid species (Harmel et al., 2008; Carolan et al., 2009; Rao et al., 2013; Yang et al., 2018). As an alternative, the comparative proteomic analysis of bamboo samples unfed and fed by *P. bambucicola* may help better determine candidate proteins secreted into hosts during natural feeding process. Functional analyses of salivary gland proteins and saliva proteins reflect important roles of salivary glands in protein secretion and energy metabolism. Although 1213 transcripts are predicted to encode putative secretory proteins, only 267 secretory proteins can be detected in the salivary gland and/or saliva proteomes of *P. bambucicola* (**Table S5**). Consistent with previous studies (Chaudhary et al., 2015; van Bel and Will, 2016; Zhang et al., 2017; Yang et al., 2018; Zhang et al., 2020), some insect detoxification enzymes, peroxidases, digestion enzymes, effectors modulating plant defenses and salivary sheath proteins can be also detected in salivary gland and/or saliva of *P. bambucicola*. Among them, the glucose dehydrogenase, glutathione S-transferase and carboxylesterases are important detoxifying enzymes used by insects to protect against plant defensive compounds (Cox-Foster and Stehr, 1994; Yu et al., 2009; Koirala et al., 2022). Peroxidases are one of the primary antioxidative enzymes of insects and may be involved in protecting *P. bambucicola* from plant oxidative damage. Salivary sugar degrading enzymes, peptidases and proteases in insects can function as important digestive enzymes degrading plant polysaccharide and plant defense proteins (Nicholson et al., 2012; Liu et al., 2016; Zhang et al., 2017). Some digestive enzymes detected in *P. bambucicola* salivary gland or saliva secretomes have been also found in salivary gland or saliva of some aphids and other phloem-feeding insects, such as the lysosomal alpha-mannosidase (Wu et al., 2021), carboxypeptidase (Huang et al., 2020), cathepsin (Foissac et al., 2002; Zhang et al., 2017; Huang et al., 2020; Wu et al., 2021; Zhang et al., 2022), serine protease (Nicholson et al., 2012; Zhang et al., 2017) and phospholipase (Zhang et al., 2017). These putative secretory effectors may be essential for enabling *P. bambucicola* feeding on bamboo host, such as helping promote aphid stylet penetration, digestion and detoxification of toxins, or suppressing plant defenses against *P. bambucicola*. In addition, some secretory proteins homologous to known aphid effectors involved in modulating plant defenses are also detected in *P. bambucicola* salivary gland and saliva, including apolipophorins (Chaudhary et al., 2015; Zhang et al., 2017; Zhang et al., 2020), odorant binding protein (Zhang et al., 2017), protein yellow (Chaudhary et al., 2015) and some salivary sheath components (van Bel and Will, 2016; Wu et al., 2021). The high similarities in the composition of salivary secretory proteins across different aphid species may highlight their importance in aphid-plant interactions. However, whether these candidate effectors of *P. bambucicola* play conserved roles in modulating aphid-plant interactions remains to be explored. The role of potentially effector proteins of unknown function in aphid-host interaction is also worth further investigation, which may provide new insight into the mechanisms of aphid’s adaption to bamboo host.

To successfully feed on the hard bamboo stalks, *P. bambucicola* must first overcome and penetrate the physical barrier of the plant cell wall. In this process, aphids require multiple PCWDEs to break down the plant cell wall polysaccharides (Silva-Sanzana et al., 2020). We identified potential PCWDEs based on transcriptomes of whole body and salivary glands, and proteomes of salivary gland and saliva of *P. bambucicola*. Although multiple transcripts of β-glucosidases, endo-β-1,4-glucanases and β-mannosidases can be identified in salivary gland transcriptome and body transcriptome for *P. bambucicola* of different morphs and developmental stages, most of identified PCWDE candidates in salivary glands show very low expression levels. And given the multiple functions of some enzymes (such as β-glucosidases) (Watanabe and Tokuda, 2010), the activities and functions of these enzymes potentially involved in degradation of cellulose and hemicellulose need to be further verified. Moreover, it seems that this aphid can only encode a small fraction of the complete set of enzymes for degrading cellulose and hemicellulose. For example, the cellulose degradation process needs the involvement of three kinds of enzymes: the endo-β-1,4-glucanase that hydrolyse cellulose randomly, exo-β-1,4-glucanase that hydrolyse cellulose from the reducing or non-reducing end to release cellobiose, and β-glucosidase cleaving cellobiose or cello-oligosaccharides into glucose monomers (Gilbert, 2010; Watanabe and Tokuda, 2010). The *P. bambucicola* seems to lack the key exo-β-1,4-glucanase that is responsible for the intermediate steps of cellulose degradation. The same case is in the hemicellulase system where *P. bambucicola* lacks the main-chain hemicellulases, such as the xylanase and xylooligosaccharidase, while β-mannosidases are only side-chain degrading enzymes that hydrolyse the hemicellulosic oligosaccharides into monomeric sugars (Tokuda, 2019). In addition, we did not found any pectinases in either salivary gland or body samples of *P. bambucicola*. Pectinases are thought to be required for aphid stylet penetration between cells (McAllan and Adams, 1961). Pectin degradation plays an important role in the degradation of plant cell wall, which can promote the further degradation of cellulose and hemicellulose and make cell wall more easily decomposed by other enzymes (Calderón-Cortés et al., 2012). Our results suggest that *P. bambucicola* itself may not have the ability to produce pectinases, thereby failing to complete even the first step of cell wall degradation. *Via* Our findings imply that *P. bambucicola* may not be able to degrade plant cell walls on its own and may require the help of its symbiotic bacteria (Liu et al., 2021; Liu et al., 2022). Further study on functional interaction between this aphid and its dominate symbiotic bacteria is especially needed.

We also investigate the response of bamboo to *P. bambucicola* feeding by comparative proteomic analysis of bamboo tissues unfed and fed by aphids. The downregulated proteins in bamboo after being fed were mainly enriched in vesicle, golgi apparatus, plant cell wall biogenesis. These findings suggest that aphid feeding may inhibit the bamboo’s normal physiological processes, such as breaking down plant cell wall and suppressing the plant cell wall synthesis activity, which may be mediated by aphid effectors secreted into host to maintain aphid’s feeding.

## Data Availability

The data presented in the study are deposited in the National Center for Biotechnology Information (NCBI) BioProject database under accession number PRJNA900789 (salivary gland) and PRJNA901050 (whole body), and the ProteomeXchange database with the dataset identifier PXD038131.
