# Supplementary material for: Transcriptomic and proteomic analyses provide insights into host adaptation of a bamboo-feeding aphid
Source: Front Plant Sci. 2023 Jan 11;13:1098751. doi: 10.3389/fpls.2022.1098751 (PMC9874943; doi:10.3389/fpls.2022.1098751)
Supplement: Supplementary file 1 [file DataSheet_1.pdf]

Supplementary Material

Supplementary Figures

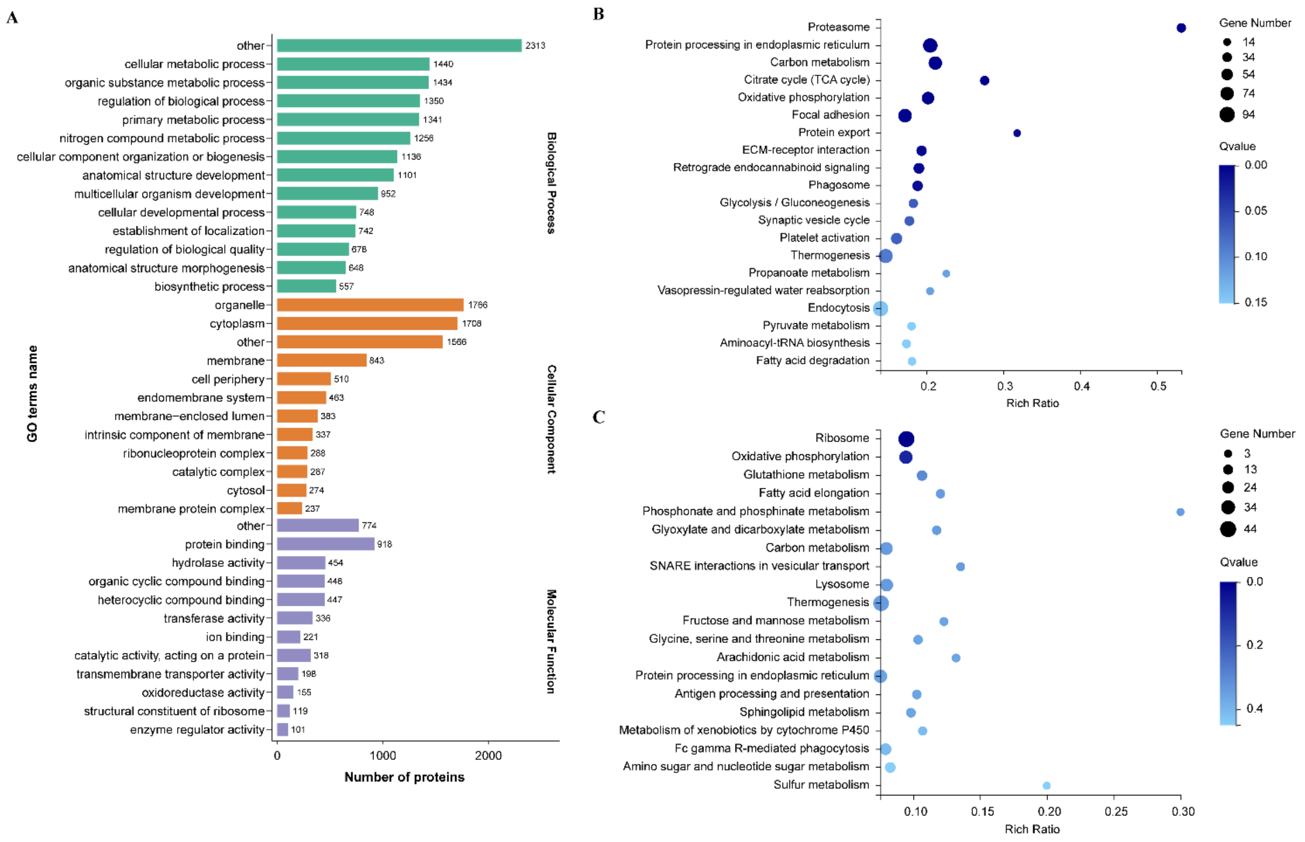

Supplementary Figure 1. Functional analysis of salivary proteins of *Pseudoregma bambucicola*. GO functions of *P. bambucicola* salivary gland proteins (A). Bubble plot of KEGG pathway enrichment for salivary gland proteins (B) and putative salivary proteins secreted into host plants (C). Only the top 20 pathways with the smallest FDR adjust *P*-value (Qvalue) were displayed.

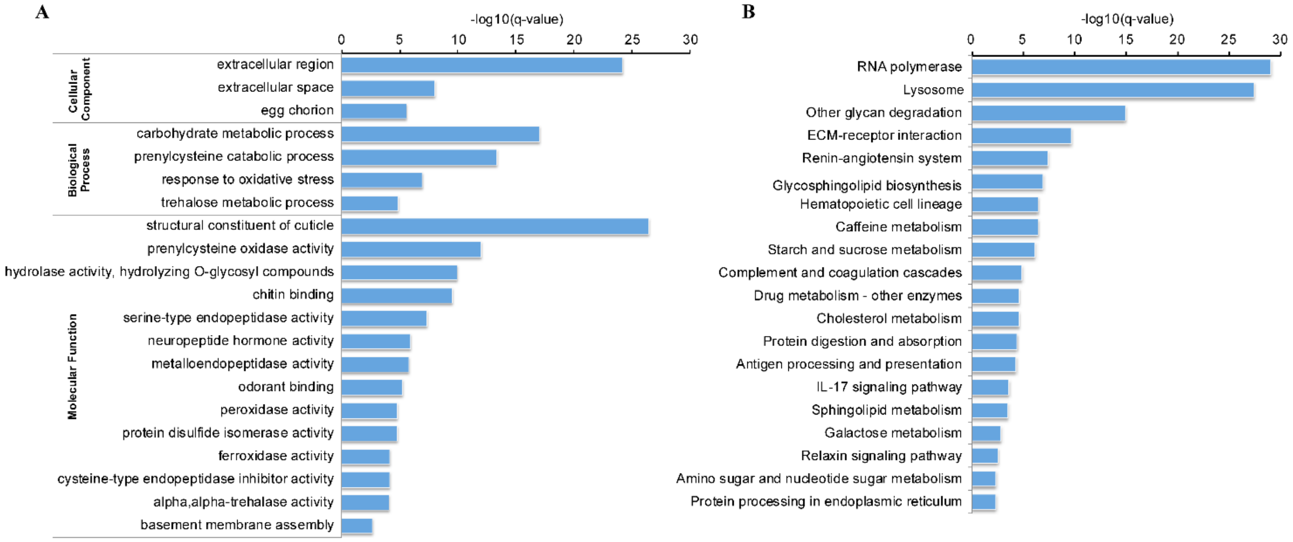

Supplementary Figure 2. Functional enrichment analysis of putative secretory proteins identified from salivary glands of *Pseudorema bambucicola*. GO term enrichment (A) and KEGG pathway enrichment (B) for salivary secretory proteins. Only the top 20 most enriched categories were displayed.

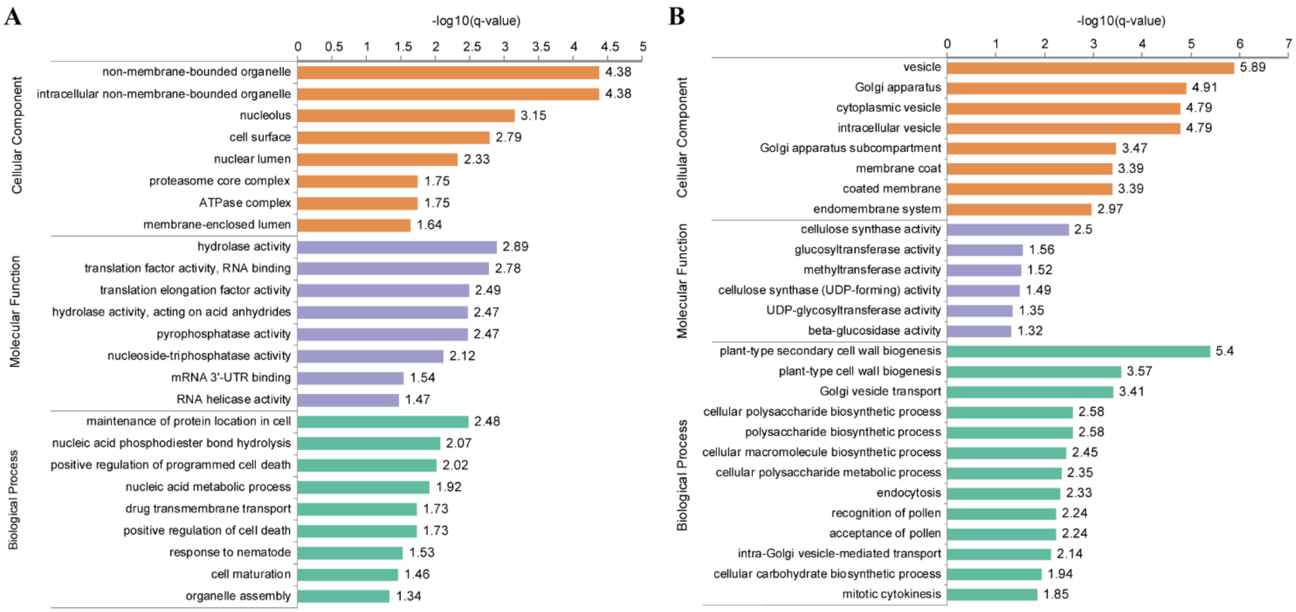

Supplementary Figure 3. Functional enrichment analysis of differentially expressed bamboo proteins between bamboos fed and unfed by *Pseudorema bambucicola*. (A) GO term enrichment of upregulated proteins (A) and downregulated proteins (B) in bamboos fed by *P. bambucicola*.
